# The relationship between myofiber characteristics and meat quality of Chinese Qinchuan and Luxi cattle

**DOI:** 10.5713/ajas.20.0066

**Published:** 2020-06-24

**Authors:** Xiao Lu, Yuying Yang, Yimin Zhang, Yanwei Mao, Rongrong Liang, Lixian Zhu, Xin Luo

**Affiliations:** 1 College of Food Science and Engineering, Shandong Agricultural University, Taian, Shandong 271018, China

**Keywords:** Beef Muscles, Meat Quality, Myofiber Characteristics

## Abstract

**Objective:**

The objectives of this study were to explore the expression patterns of myosin heavy chain (*MyHC*) genes of different skeletal muscles from Chinese cattle, and to investigate the relationship between myofiber characteristics and meat quality of *M. longissimus lumborum* (LL), *M. psoas major* (PM), and *M. semimembranosus* (SM) from Chinese Luxi and Qinchuan cattle.

**Methods:**

Three major muscles including LL, PM, and SM from Chinese Luxi cattle and Chinese Qinchuan cattle were used in this study. The myofiber characteristics were measured by histochemical analysis. The MyHC isoforms expression was evaluated by real-time quantitative polymerase chain reaction. Quality traits including pH value, meat color, cooking loss, Warner-Bratzler shear force (WBSF) and sarcomere length were determined at day 5 postmortem.

**Results:**

PM muscle had higher pH value, a* value, sarcomere length and lower WBSF value compared to LL and SM muscles (p<0.05). Numbers of type I myofiber and the relative expression of *MyHC I* mRNA in PM muscle were higher than those of LL and SM muscles (p<0.05). Myofiber diameter of PM muscle was lower than that of LL and SM muscles, regardless of myofiber types (p<0.05).

**Conclusion:**

According to the stepwise linear regression analyses, tenderness was influenced by myofiber characteristics in all three examined muscles. Tenderness of beef muscles from Qinchuan and Luxi cattle could be improved by increasing numbers of type I myofiber.

## INTRODUCTION

Breed is an important factor that can influence the characteristics of meat products. Luxi and Qinchuan cattle are two famous indigenous Chinese breeds widespread throughout the central area of China. Many local consumers believe that beef from native cattle possess better meat quality than imported beef. Therefore, higher price has been considered for beef from Luxi and Qinchuan cattle in central China.

Meat quality traits including tenderness, color and water holding capacity (WHC) are always crucial to consumers and consequently to the beef industry [1]. It is well known that beef quality of different muscle sources is highly variable, and numerous factors can be responsible for the variations of these quality characteristics [2–5].

Myofiber characteristics largely affect meat quality because muscle fiber is a primary component of skeletal muscle. Myofiber can be categorized by their morphological traits, contractile and metabolic properties [6]. Based on the histochemical reaction for myosin adenosine triphosphatase (mATPase), myofibers have been divided into three types, including slow-oxidative or type I, fast oxido-glycolytic or type IIa, and fast glycolytic IIb [7]. The distribution of myosin heavy chain (MyHC) based myofiber typing is another useful method for classification of myofibers [8]. There are eight isoforms of MyHC in mammalian skeletal muscles, but only *MyHC I*, *MyHC IIa*, and *MyHC IIx* were generally expressed in adult bovine skeletal muscle [9]. Previous studies have outlined that the expression patterns of *MyHC* genes varied between bovine muscles and cattle breeds [10–12]. Thus, further research is needed to elucidate the mechanism on *MyHC* genes expression of different skeletal muscles from Chinese native cattle.

Several studies have investigated the relationship between myofiber characteristics and meat quality of individual beef muscles [4,13,14]. However, the magnitude of their influences on beef quality is still controversial. Moreover, little information is available on the relationship between myofiber characteristics and meat quality of Chinese indigenous cattle. Therefore, the objectives of this study were to explore the expression patterns of *MyHC* genes of different skeletal muscles from Chinese Luxi and Qinchuan cattle, and to investigate the relationship between myofiber characteristics and meat quality of individual muscles from these cattle.

## MATERIALS AND METHODS

### Sample preparation

A total of 20 Luxi and Qinchuan bulls were randomly selected at a commercial abattoir. Ten animals from each breed were slaughtered at 24 months of age, according to the National Standard of China (GB/T 19477-2004). Carcasses were not electrically stimulated. Within 45 min postmortem, two pieces of 5 g samples from three major muscles including *longissimus lumborum* (LL), *psoas major* (PM), and *semimembranosus* (SM) were taken and frozen using liquid nitrogen for histochemical analysis and *MyHC* gene expression. At 48 h postmortem, all the muscles were collected from the carcasses, individually vacuum packaged and transferred on ice to the laboratory. Three pieces of 2.54 cm thick steak were obtained from central part of each muscle upon arrival. Quality traits including pH value, meat color, cooking loss, Warner-Bratzler shear force (WBSF) and sarcomere length were determined.

### Histochemical analyses

Histochemical analyses were carried out by the method described by Brooke and Kaiser [15]. Cross sections (10 μm) were obtained using a cryostat (Cryostar NX50, Thermo Scientific, Waltham, MA, USA) at −20°C. The sections were subsequently used for histochemical analyses of mATPase following alkaline preincubation. This technique consisted of the preparation of three initial solutions: solution A, Tris-base (5 g) and CaCl_2_ (0.5 g) dissolved in 250 mL distilled water; solution B, the pH value of solution A adjusted to 10.4 by HCL; solution C, the pH value of Solution A adjusted to 9.4 by ATP. Samples were incubated in solution B for 5 min and solution C for 30 min. The next steps consisted of washing samples three times with solution A for 2 min each; application of Co(NO_3_)_2_ on the cross section for 5 min; rinsing of samples with distilled water; addition of ammonium sulfide solution (1%) for 30 seconds. Images were captured by a microscope (Eclipse E100, Nikon, Tokyo, Japan). The stained sections were examined using an image analysis system (ImagePro plus 6.0, Media Cybernetics, Rockville, MD, USA). Approximately 300 myofibers per sample were counted to analyze the myofiber characteristics. Myofiber type, myofiber number percentage, myofiber diameter, and myofiber area percentage were determined.

### Quantification of mRNA

Total RNA was extracted using RNAiso Plus reagent (TaKaRa, Beijing, China) according to the manufacturer’s protocol. Then, genomic DNA was removed from total RNA sample. Subsequently, the purity and integrity of RNA were evaluated by optical density under 260 nm and 280 nm wavelength and by 1% agarose gel electrophoresis, respectively. Reverse transcription of mRNA was done with a PrimeScript RT reagent Kit with gDNA Eraser (TaKaRa, China). The cDNA was stored in a refrigerator at −20°C for real-time quantitative polymerase chain reaction (qRT-PCR). Quantification of mRNA was performed with a real-time PCR system (CFX96, Bio-Rad, Hercules, CA, USA) with SYBR Premix Ex *Taq* (TaKaRa, China). In this study, glyceraldehyde-3-phosphate dehydrogenase (*GAPDH*) was selected as the reference gene. The primer sequences are shown in Table 1. Relative gene expression was calculated by 2^−ΔCt^ according to the method of Zhang et al [9].

### Meat quality traits

A portable pH meter with temperature compensation (SenvenGo, Mettler Toledo, Zurich, Switzerland) was used to determine pH value. The pH electrode was calibrated in buffers at pH 4.00 and 7.00 before use at room temperature. Measurements were taken three times in different locations for each sample.

Surface color was measured using a portable colorimeter (SP62, X-Rite, Grand Rapids, MI, USA) with 4 mm diameter aperture, Illuminant A, 10° observer angle. The instrument was calibrated with a white and a black standard plate before use. Steaks were exposed to the air at room temperature for 30 min blooming before measurements. Each sample was measured at 6 different locations.

The measurement of cooking loss was performed accord ing to Lu et al [16]. Steaks were cooked individually in a water bath at 80°C until the core temperature reached 70°C. During heating, a digital thermometer was inserted into each sample to track the core temperature. The cooked steaks were cooled to the room temperature and stored at 4°C overnight. Cooking loss was expressed as the percentage of weight loss before and after heating.

WBSF was determined as described by Lu et al [16]. After measuring cooking loss, six columnar samples with the diameter of 1.27 cm were removed parallel to the myofibers from each steak. Each sample was sheared once perpendicular to the fiber orientation using a texture analysis machine (TA-XT2i, Stable Micro Systems, Godalming, UK) with a HDP/BSW blade. The average measurement of columnar samples from each steak was the value of WBSF in Newtons (N).

Sarcomere length was measured according to the method of Li et al [17] with minor modifications. Four grams of beef muscle was mixed with 35 mL of 0.25 M sucrose solution for 1 min in a homogenizer (T18, IKA, Staufen, Germany) at low speed. Then a drop of the slurry containing the fiber fragments was transferred on a microscopic slide and covered with a cover slip. The suspension was examined with the oil objective under a phase-contrast microscopy (BX41, Olympus, Tokyo, Japan), and 30 single myofibrils were photographed by an Olympus camera. All the images were analyzed by the software Image-Pro Plus (6.0, Media Cybernetics, USA). Five measurements of sarcomere length were performed at different points on each image. The average of 150 measurements was designated as the sarcomere length of one sample.

### Statistical analysis

The experimental data were analyzed by the MIXED procedure of Statistical Analysis System (SAS, Version 9.2, Cary, NC, USA). The MIXED procedure was used with cattle breed, muscle type, and their interaction as fixed factors, and animal as a random factor. Tests of differences between predicted means were applied using the PDIFF statement and differences were considered significantly different at p<0.05. To analyze the relationship between myofiber characteristics and meat quality, stepwise multiple linear regression was used (SPSS, Version 18.0, Chicago, IL, USA).

## RESULTS AND DISCUSSION

### Myofiber characteristics

**Figure 1:**
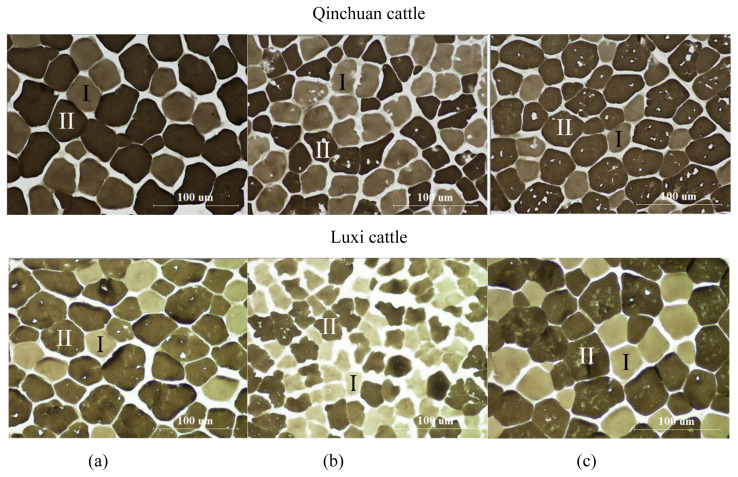
Images of myosin ATPase staining (pH 9.4) of (a) *M. longissimus lumborum*, (b) *M. psoas major* (c) *M. semitendinosus* muscles.

The myofiber types in three different muscles obtained from Qinchuan and Luxi cattle were divided into type I and type II, based on alkaline m-ATPase reactions (Figure 1). A clear difference in myofiber type composition was observed among three muscles. PM muscle had a distinguishably higher number percentage of myofiber type I compared with LL and SM muscles (Table 2). This is consistent with Hwang et al [13] and Lang et al [14], who reported a higher proportion of type I myofiber in PM muscle than LL muscle. There were no differences for myofiber number percentage between Qinchuan and Luxi cattle. Ryu et al [18] concluded that muscle location was more important than breed in affecting myofiber type composition, which is in support of our study.

**Figure 2:**
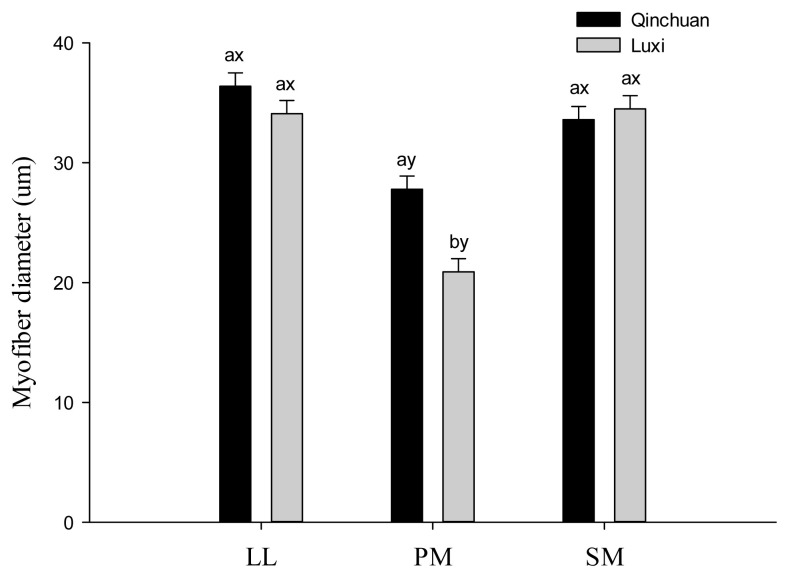
Myofiber diameter of 3 major muscles in Chinese Qinchuan and Luxi cattle. LL, *longissimus lumborum*; PM, *psoas major*; SM, *semimembranosus*. ^a,b^ Means within the same muscle with different letters differ (p<0.05); ^x,y^ Means within the same breed with different letters differ (p<0.05).

Results of the myofiber diameter are presented in Figure 2. There was a significant interaction effect of cattle breed× muscle type on the diameter of myofiber (p<0.05). Joo et al [1] reported that myofiber diameter varies with species, chronological age, nutritional state of the animal, genetic background, and composition of myofibers. As expected, the diameter of myofibers in LL and SM muscles were significantly longer than PM muscle (p<0.05). This finding is in line with Joo et al [4], who also observed smaller myofiber diameter in PM muscle than LL and SM muscles. There was no difference between LL and SM muscle for the myofiber diameter, irrespective of cattle breeds. However, PM muscle from Qinchuan cattle exhibited larger myofiber diameter than that of Luxi breed (p<0.05).

Differences in myofiber area percentage are shown in Table 3. Myofiber type, breed and their interaction had significant effect on myofiber area percentage (p<0.05). The area percentage of myofiber did not differ between Qinchuan cattle and Luxi cattle. However, the area percentage of type I myofiber was significantly lower than that of type II myofiber in both cattle breeds (p<0.05), which is similar with the results of Joo et al [4].

### Expression pattern of myosin heavy chain isoforms

As shown in Table 4, the expression patterns of *MyHC I*, *MyHC IIa*, and *MyHC IIx* were compared. The mRNA abundances of *MyHC I* were significantly higher than *MyHC IIa* and *MyHC IIx* (p<0.05), irrespective of cattle breeds. The expression level of *MyHC I* in Qinchuan cattle was higher than that of Luxi cattle (p<0.05). However, there were no difference in the expression level of *MyHC IIa* and *MyHC IIx* mRNA between different cattle breeds (p>0.05). The expressions level of *MyHC I* in PM muscle was distinctly higher than LL and SM muscles (p<0.05), which is consistent with the histochemical analyses above. In the present study, *MyHC I*, *MyHC IIa*, and *MyHC IIx* were expressed in bovine LL, PM, and SM muscles, whereas *MyHC IIb* expression was not detected. Zhang et al [9] and Oe et al [19] similarly reported the absence of *MyHC IIb* expression in bovine skeletal muscles. A previous study concluded that *MyHC IIb* expression was scarcely found in most cattle breeds except for young Blond d’Aquitaine bulls, and the *MyHC IIb* transcript was not translated in all beef muscles [11]. However, skeletal muscles from small rodent animals have been observed to express the four major MyHC isoforms [20]. Therefore, the expression of *MyHC IIb* gene was muscle-specific and species-specific.

### Meat quality traits

In this study, the main effects of cattle breed and muscle type significantly affected pH values (p<0.05). As shown in Table 5, all the muscles had a normal pH value ranging from 5.46 to 5.68. Steaks from Qinchuan cattle possessed a higher pH value than that of Luxi cattle (p<0.05). In addition, the pH value of PM muscle was higher than that of LL and SM muscles (p<0.05). This finding is consistent with Liu et al [21], who also reported a higher ultimate pH of PM muscle compared to LL muscle from Chinese Luxi cattle.

Meat color is an important factor influencing purchase decision. Consumers usually identify bright-red colored meat as evidence of freshness and wholesomeness at the point of sale [22]. L* values of three different muscles were only affected by cattle breed (p<0.05). There was no significant interaction effect of breed×muscle for the L* and b* values (p>0.05). As shown in Table 5, steaks from Qinchuan cattle had higher L* values than that of Luxi breed. However, the present study did not observe any variation for the L* values among different muscle types. Kim et al [23] similarly reported no significant differences in lightness values among different bovine muscles. Muscle type, cattle breed and their interactions significantly affected the a* values (p<0.05). Redness values of steaks from both Qinchuan and Luxi cattle were significantly higher in PM and SM muscles compared with LL muscle (p<0.05). Myoglobin content is a crucial factor affecting a* values in postmortem muscles. In the present study, a higher percentage of type I myofiber was observed in PM muscle, demonstrating a higher myoglobin content in PM muscle. Thus, a higher a* values of PM muscle was well explained. In addition, LL muscle from Qinchuan cattle had a higher a* values compared to Luxi cattle (p<0.05). b* values were only affected by muscle type (p<0.05). PM and SM muscles exhibited higher b* values than that of LL muscle.

Cooking loss is an important parameter to reflect WHC of beef muscle. There was a significant interaction effect of breed×muscle for cooking loss (p<0.05). SM muscle showed the highest cooking loss compared to LL and PM muscles (Table 5), which is in accordance with the results of Joo et al [4].

Tenderness is one of the most important attributes re garding eating quality [24]. Muscle type, cattle breed and their interactions had significant effect on WBSF value and sarcomere length (p<0.05). As shown in Table 5, the lowest WBSF value along with longest sarcomere length was observed in PM muscles, which is consistent with the findings of Joo et al [4] and Hwang et al [13]. In addition, the WBSF values of LL muscle was higher than that of PM and SM muscles (p<0.05). This is partly in agreement with previous studies [25,26]. Variations in the structure, composition, solubility of intramuscular connective tissues and sarcomere length are known to exist between different muscle types and are related to variations in tenderness [5].

### Relationships between myofiber characteristics and meat quality traits

It is generally accepted that muscle’s metabolic properties affected by total number of myofibers, cross-sectional area of myofibers and myofiber type composition greatly influence meat quality [1]. Considering the variations in meat quality among LL, PM, and SM muscles from Qinchuan and Luxi cattle, the regression analyses were conducted between meat quality parameters and myofiber characteristics.

Previous studies reported that muscle pH _24_ was negatively correlated with the percentage of myofiber type IIa [13,27]. If type II myofibers are predominant in the muscle, postmortem glycolysis is rapidly induced, resulting in an accelerated pH decline in the muscle [28]. However, our present study showed no significant correlation between pH values and myofiber characteristics, probably due to the delayed pH measurement.

It is well documented that the a* value and myoglobin content increased with a higher proportion of red myofiber, which decreased color stability with a possible shift to a brownish metmyoglobin color [1]. Ryu and Kim [29] reported that muscle with a higher percentage of type I and type IIa myofiber had lower L* values. However, Hwang et al [13] reported that there was no significant correlation between the a* value and myofiber types, which is similar to the results in this study. There is evidence that an incompatibility of the mATPase based and MyHC-based myofiber classification may exist [30]. It is difficult to differentiate pure myofiber types such as IIa and IIx from hybrid myofibers by using histochemical reaction intensity. In the present study, the myofiber were classified only based on the contractile property, i.e., slow (type I) and fast myofibers (type II). Thus, the correlations between pH values, meat color, cooking loss and myofiber characteristics were not significant (p>0.05).

Significant correlations were observed between tenderness and myofiber characteristics in all three muscles from both cattle breeds. Ozawa et al [27] and Ryu et al [29] reported positive correlation between myofiber type I and shear force and negative correlation between type IIb and shear force. However, the relationship between myofiber type composition and meat tenderness is still under debate. Hwang et al [13] found that myofiber type I was negatively correlated with WBSF. The same correlation was also observed in the present study. To predict the tenderness using myofiber characteristics, a multiple linear regression analysis was conducted by a stepwise method (Table 6). A linear regression equation for WBSF value was deduced as Y_1_ (WBSF value) = 55.45+0.74X_1_ (the diameter of myofiber type I)–0.52X_2_ (the number percentage of myofiber type I), Y_2_ (WBSF value) = 1.62+0.82 X_3_ (the diameter of myofiber type II)+0.45 X_4_ (the number percentage of myofiber type II). The diameter and the number percentage of myofiber are the major elements determining the WBSF value, which can explain 42% and 44% of the total model, respectively. When the diameter of myofiber type I and II increased by 1 unit, the WBSF value increased by 0.74 and 0.82, respectively. When the number percentage of myofiber type I and II increased by 1 unit, the WBSF value decreased by 0.52 and increased by 0.45, respectively.

Similarly, a linear regression equation for sarcomere length was deduced as Y_3_ (sarcomere length) = 4.34–0.08X_1_ (the diameter of myofiber type I), Y_4_ (sarcomere length) = 4.57–0.07X_3_ (the diameter of myofiber type II). The diameters of myofiber are the major elements determining the sarcomere length, which can explain 57% and 55% of the model, respectively. When the diameter of myofiber type I and II increased by 1 unit, the sarcomere length decreased by 0.08 and 0.07, respectively. Therefore, it is possible to quantitatively predict the tenderness using these parameters. Consequently, these findings suggest that beef tenderness could be improved by increasing type I myofiber.

## CONCLUSION

A clear difference in meat quality and myofiber type composition was observed between LL, PM, and SM muscles from Chinese Qinchuan and Luxi cattle. Among the muscles, PM showed the highest a* value and lowest WBSF value. In addition, PM muscle had the highest proportion of type I myofiber and abundances of mRNA of *MyHC I*. SM muscle exhibited the highest composition of type II myofiber, along with higher WBSF value. The significant differences in myofiber characteristics may influence beef tenderness. Hence, it is suggested that myofiber characteristics of beef muscle could be an important indicator of tenderness.

## Figures and Tables

**Table 1:** Primer sequences

Genes	Primer sequence (5’→3′)	Product size (bp)
*GAPDH*	F:GATGGTGAAGGTCGGAGTGAAC	100
	R:GTCATTGATGGCGACGATGT	100
*MyHC I*	F:ATCGCTGAATCCCAGGTCAA	92
	R:ACCAAGATGTGGCACGGCTA	92
*MyHC IIa*	F:CACCCTGGAGCAGACAGAGA	148
	R:TCCCTGGATTTGCGTGATG	148
*MyHC IIx*	F:TTTCCAGACCGTGTCTGCTC	96
	R:GGGATGATGCAGCGTACAAAG	96
*MyHC IIb*	F:AGGGCATCGCTGGAACAGAC	84
	R:CAGAAGCTGCACACGCTCAC	84

*GAPDH*, glyceraldehyde-3-phosphate dehydrogenase; *MyHC*, myosin heavy chain.

**Table 2:** Fiber number percentage of 3 major muscles from Chinese Qinchuan and Luxi cattle (%)

Cattle breed	Fiber type	Muscle type			SE

		LL	PM	SM	
Qinchuan	I	27.7^b^^y^	42.0^a^^y^	25.4^b^^y^	2.4
	II	72.3^a^^x^	58.0^b^^x^	74.6^a^^x^	
Luxi	I	22.8^c^^y^	45.0^a^^y^	34.2^b^^y^	
	II	77.2^a^^x^	55.0^c^^x^	65.8^b^^x^	

LL, *M. longissimus lumborum*; PM, *M. psoas major*; SM, *M. semimembranosus*; SE, standard error.

a–c Different letters indicate significant differences between muscle types for the same cattle breed and fiber type (p<0.05).

x,y Different letters indicate significant differences between fiber types for the same cattle breed and muscle type (p<0.05).

**Table 3:** Fiber area percentage of 3 major muscles from Chinese Qinchuan and Luxi cattle (%)

Cattle breed	Fiber type	Muscle type			SE

		LL	PM	SM	
Qinchuan	I	39.2^x^	42.2^x^	40.6^x^	1.4
	II	60.8^y^	57.8^y^	59.4^y^	
Luxi	I	40.1^x^	37.4^x^	39.7^x^	
	II	59.9^y^	62.6^y^	60.3^y^	

LL, *M. longissimus lumborum*; PM, *M. psoas major*; SM, *M. semimembranosus*; SE, standard error.

x,y Different letters indicate significant differences between fiber types for the same cattle breed and muscle type (p<0.05).

**Table 4:** Expression levels of *MyHC* genes in 3 major muscles from Chinese Qinchuan and Luxi cattle

Cattle breed	Genes	Muscle type			SE

		LL	PM	SM	
Qinchuan	*MyHC I*	9.5^a^^x^	11.9^a^^x^	7.5^a^^x^	0.96
	*MyHC IIa*	2.7^a^^y^	2.8^a^^y^	2.3^a^^y^	
	*MyHC IIx*	2.0^a^^y^	2.3^a^^y^	2.3^a^^y^	
Luxi	*MyHC I*	3.8^b^^x^	8.8^a^^x^	4.3^a^^x^	
	*MyHC IIa*	3.2^a^^x^	2.3^a^^y^	5.3^a^^x^	
	*MyHC IIx*	2.9^a^^x^	2.5^a^^y^	3.2^a^^x^	

*MyHC*, myosin heavy chain; LL, *M. longissimus lumborum*; PM, *M. psoas major*; SM, *M. semimembranosus*; SE, standard error.

a,b Different letters indicate significant differences between cattle breeds for the same gene and muscle type.

x,y Different letters indicate significant differences between genes for the same cattle brees and muscle type (p<0.05).

**Table 5:** Meat quality traits for 3 major muscles from Chinese Qinchuan and Luxi cattle

Variables	Breed	Muscle			SE	Mean±SE	p-value		
	
		LL	PM	SM			Breed	Muscle	Muscle×breed
pH	QC	5.61	5.68	5.63	0.03	5.64±0.02^a^	***	*	NS
	LX	5.50	5.55	5.46		5.50±0.02^b^			
	Mean	5.55^y^	5.62^x^	5.54^y^	0.02				
L*	QC	41.0	44.5	43.5	0.9	43.0±0.5^a^	**	NS	NS
	LX	40.4	40.8	40.0		40.4±0.5^b^			
	Mean	40.7	42.6	41.8	0.7				
a*	QC	18.5^a^^y^	20.2^a^^x^	19.7^a^^x^	0.5	19.5±0.3	***	***	**
	LX	16.3^b^^y^	20.4^a^^x^	19.6^b^^x^		18.8±0.3			
	Mean	17.4	20.3	19.7	0.3				
b*	QC	14.7	16.8	17.9	0.5	16.5±0.3	NS	***	NS
	LX	14.9	17.2	17.1		16.4±0.3			
	Mean	14.8^y^	17.0^x^	17.5^x^	0.4				
Cooking loss (%)	QC	20.3^b^^z^	26.3^a^^y^	30.9^a^^x^	0.6	25.8±0.3	NS	***	**
	LX	23.0^a^^y^	24.7^a^^y^	28.6^b^^x^		25.4±0.3			
	Mean	21.6	25.5	29.7	0.4				
WBSF (N)	QC	77.2^a^^x^	42.6^a^^y^	72.9^a^^x^	2.0	64.2±1.1	***	***	***
	LX	62.2^b^^x^	46.6^a^^z^	55.8^b^^y^		54.9±1.1			
	Mean	69.7	44.6	64.3	1.4				
Sarcomere length (μm)	QC	1.7^a^^y^	2.3^b^^x^	1.8^b^^y^	0.1	1.9±0.1	***	***	***
	LX	1.9^a^^y^	3.4^a^^x^	2.1^a^^y^		2.5±0.1			
	Mean	1.8	2.8	1.9	0.1				

LL, *M. longissimus lumborum*; PM, *M. psoas major*; SM, *M. semimembranosus*; SE, standard error; WBSF, Warner-Bratzler shear force; QC, Qinchuan cattle; LX, Luxi cattle.

a,b Means with different superscript letters within the same columns differ at p<0.05.

x–z Means with different superscript letters within the same rows differ at p<0.05.

NS, not significant;

* p<0.05;

** p<0.01;

*** p<0.0001.

**Table 6:** Regression equation and coefficient between meat quality traits and myofiber characteristics of Chinese Qinchuan and Luxi cattle

Type	Dependent variable	Adj-R^2a^	Independent variable	Regression coefficient	t value	p-value
I	WBSF	0.42***	Myofiber diameter	0.74	2.36	0.022
			FNP	−0.52	−2.79	0.008
	Sarcomere length	0.57***	Myofiber diameter	−0.08	−8.01	0.000
II	WBSF	0.44***	Myofiber diameter	0.82	2.72	0.009
			FNP	0.45	2.36	0.023
	Sarcomere length	0.55***	Myofiber diameter	−0.07	−7.69	0.000

WBSF, Warner-Bratzler shear force; FNP, fiber number percentage.

*** p<0.0001.

## References

[1] Joo ST, Kim GD, Hwang YH, Ryu YC (2013). Control of fresh meat quality through manipulation of muscle fiber characteristics. Meat Sci.

[2] McKenna DR, Mies PD, Baird BE, Pfeiffer KD, Ellebracht JW, Savell JW (2005). Biochemical and physical factors affecting discoloration characteristics of 19 bovine muscles. Meat Sci.

[3] Stolowski GD, Baird BE, Miller RK (2006). Factors influencing the variation in tenderness of seven major beef muscles from three Angus and Brahman breed crosses. Meat Sci.

[4] Joo SH, Lee KW, Hwang YH, Joo ST (2017). Histochemical characteristics in relation to meat quality traits of eight major muscles from Hanwoo steers. Korean J Food Sci Anim Resour.

[5] Nair MN, Canto AC, Rentfrow G, Suman SP (2019). Muscle-specific effect of aging on beef tenderness. LWT.

[6] Lee SH, Joo ST, Ryu YC (2010). Skeletal muscle fiber type and myofibrillar proteins in relation to meat quality. Meat Sci.

[7] Choi YM, Kim BC (2009). Muscle fiber characteristics, myofibrillar protein isoforms, and meat quality. Livest Sci.

[8] Kim GD, Yang HS, Jeong JY (2016). Comparison of characteristics of myosin heavy chain-based fiber and meat quality among four bovine skeletal muscles. Korean J Food Sci Anim Resour.

[9] Zhang M, Liu Y, Fu C (2014). Expression of *MyHC* genes, composition of muscle fiber type and their association with intramuscular fat, tenderness in skeletal muscle of *Simmental* hybrids. Mol Biol Rep.

[10] Chikuni K, Muroya S, Nakajima I (2004). Myosin heavy chain isoforms expressed in bovine skeletal muscles. Meat Sci.

[11] Picard B, Cassar-Malek I (2009). Evidence for expression of IIb myosin heavy chain isoform in some skeletal muscles of Blonde d’Aquitaine bulls. Meat Sci.

[12] Tanabe R, Muroya S, Chikuni K (1998). Sequencing of the 2a, 2x, and slow isoforms of the bovine myosin heavy chain and the different expression among muscles. Mamm Genom.

[13] Hwang YH, Kim GD, Jeong JY, Hur SJ, Joo ST (2010). The relationship between muscle fiber characteristics and meat quality traits of highly marbled Hanwoo (Korean native cattle) steers. Meat Sci.

[14] Lang YM, Wang YF, Sun BZ (2017). Myofiber characteristics and eating quality of three major muscles from Chinese Simmental cattle. Can J Anim Sci.

[15] Brooke MH, Kaiser KK (1970). Muscle fiber types: how many and what kind?. Arch Neurol.

[16] Lu X, Zhang Y, Zhu L, Luo X, Hopkins DL (2019). Effect of superchilled storage on shelf life and quality characteristics of *M. longissimus lumborum* from Chinese Yellow cattle. Meat Sci.

[17] Li K, Zhang Y, Mao Y (2012). Effect of very fast chilling and aging time on ultra-structure and meat quality characteristics of Chinese Yellow cattle *M. Longissimus lumborum*. Meat Sci.

[18] Ryu YC, Choi YM, Lee SH (2008). Comparing the histochemical characteristics and meat quality traits of different pig breeds. Meat Sci.

[19] Oe M, Ojima K, Nakajima I, Chikuni K, Shibata M, Muroya S (2016). Distribution of tropomyosin isoforms in different types of single fibers isolated from bovine skeletal muscles. Meat Sci.

[20] Schiaffino S, Reggiani C (2011). Fiber types in mammalian skeletal muscles. Physiol Rev.

[21] Liu C, Zhang Y, Yang X (2014). Potential mechanisms of carbon monoxide and high oxygen packaging in maintaining color stability of different bovine muscles. Meat Sci.

[22] Suman SP, Hunt MC, Nair MN, Rentfrow G (2014). Improving beef color stability: practical strategies and underlying mechanisms. Meat Sci.

[23] Kim YH, Keeton JT, Smith SB, Berghman LR, Savell JW (2009). Role of lactate dehydrogenase in metmyoglobin reduction and color stability of different bovine muscles. Meat Sci.

[24] Kemp CM, Sensky PL, Bardsley RG, Buttery PJ, Parr T (2010). Tenderness-an enzymatic view. Meat Sci.

[25] Hildrum KI, Rødbotten R, Høy M, Berg J, Narum B, Wold JP (2009). Classification of different bovine muscles according to sensory characteristics and Warner Bratzler shear force. Meat Sci.

[26] Veiseth-Kent E, Pedersen ME, Rønning SB, Rødbotten R (2018). Can postmortem proteolysis explain tenderness differences in various bovine muscles?. Meat Sci.

[27] Ozawa S, Mitsuhashi T, Mitsumoto M (2000). The characteristics of muscle fiber types of *longissimus thoracis* muscle and their influences on the quantity and quality of meat from Japanese Black steers. Meat Sci.

[28] Choe JH, Choi YM, Lee SH (2008). The relation between glycogen, lactate content and muscle fiber type composition, and their influence on postmortem glycolytic rate and pork quality. Meat Sci.

[29] Ryu YC, Kim BC (2005). The relationship between muscle fiber characteristics, postmortem metabolic rate, and meat quality of pig *longissimus dorsi* muscle. Meat Sci.

[30] Francisco CL, Jorge AM, Dal-Pai-Silva M, Carani FR, Cabeço LC, Silva SR (2011). Muscle fiber type characterization and myosin heavy chain (MyHC) isoform expression in Mediterranean buffaloes. Meat Sci.

